# Conservative Management of Muscle Invasive Bladder Cancer in Kidney-Pancreas Transplant Patient

**DOI:** 10.1155/2022/5373414

**Published:** 2022-05-29

**Authors:** Logan D. Glosser, Brandon S. Zakeri, Conner V. Lombardi, Obi O. Ekwenna

**Affiliations:** ^1^College of Medicine, University of Toledo College of Medicine and Life Sciences, USA; ^2^Department of Urology and Transplantation, University of Toledo Medical Center, USA

## Abstract

*Introduction*. Solid organ transplant increases the risk for muscle-invasive bladder cancer (MIBC). Although a common tumor, urothelial cell carcinoma (UCC) of the bladder in patients with kidney-pancreas transplants is scarcely reported. *Case Presentation*. A 65-year-old male with history of type 1 diabetes and a 14-year status post deceased donor pancreas-kidney transplant presented with 3 weeks of gross hematuria. CT scan showed multiple bladder masses. Transurethral resection of bladder tumor (TURBT) showed papillary UCC. 5 months later, the patient reported new-onset gross hematuria. TURBT showed MIBC. The patient elected for bladder-preserving TMT. On cystoscopy there was no gross evidence of carcinoma at 3.5 years of follow up. *Discussion.* Currently, no specific management guidelines target this population with MIBC. The first-line treatment for MIBC is radical cystectomy (RC) with neoadjuvant chemotherapy. For patients that are medically unfit or unwilling to undergo RC, trimodal therapy (TMT) is an alternative. TMT for bladder cancer consists of complete tumor resection with chemotherapy and radiation. This report demonstrates a unique case of a patient with kidney-pancreas transplant diagnosed with MIBC treated with TMT that has no evidence of gross tumorigenesis at 3.5 years after diagnosis. Our findings suggest that trimodal therapy should be considered for treatment of MIBC in patients with kidney-pancreatic transplants to preserve the donated allografts.

## 1. Introduction

Bladder cancer is rare in pancreatic-kidney transplant recipients, with only a few cases reported [1]. Currently, no specific management guidelines exist for transplanted patients with bladder cancer [1–3]. Bladder cancer typically presents in early stages without muscle involvement and is treated with transurethral resection of the bladder tumor (TURBT) ± intravesical chemotherapy [4]. For those with muscle-invasive bladder cancer (MIBC), the standard of care involves more aggressive treatment with radical cystectomy (RC) and neoadjuvant chemotherapy [5]. For patients unfit or unwilling to undergo RC, bladder-preserving trimodal therapy (TMT) with maximal TURBT, chemotherapy, and radiation is an alternative [5]. This report illustrates a unique case of MIBC in a patient with history of simultaneous pancreas-kidney (SPK) transplant treated with TMT. This case report has been reported in line with the SCARE criteria [6].

## 2. Case Description

A 65-year-old man with history significant for type 2 diabetes mellitus, hypertension, and ESRD with subsequent deceased donor SPK transplant 13 years prior presented with 3 weeks of gross hematuria and ongoing painful ejaculation. His other comorbidities include benign prostatic hyperplasia, dyslipidemia, and hypothyroidism. Immunosuppressive therapy included sirolimus and tacrolimus.

**Figure 1:**
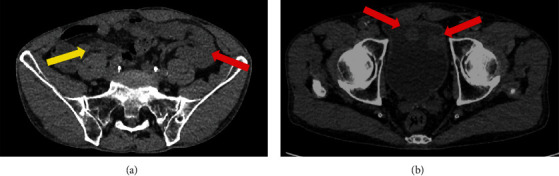
CT scan with contrast 2017. (a) Prior kidney transplant (red) and pancreas transplant (yellow). (b) Multiple masses in the bladder.

**Figure 2:**
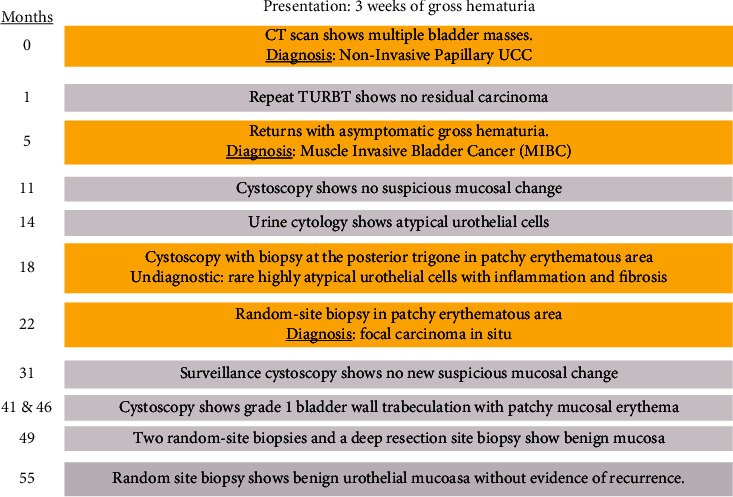
Chronologic timeline from presentation to 4 years status post muscle invasive bladder cancer treatment.

Workup with computed tomography (CT) scan of the abdomen and pelvis showed the transplanted kidney and pancreas (Figure 1(a)) and multiple masses in the bladder (Figure 1(b)). Cystoscopy revealed several sessile masses suspicious for UCC. At time zero (Figure 2), the patient was treated with TURBT and instillation of mitomycin C. The total tumor burden was greater than 10 cm. Pathology results showed high-grade T1 papillary UCC without muscle invasion. One month later, repeat TURBT demonstrated no residual carcinoma. Patient elected for follow-up and declined intravesical BCG treatment.

5 months post-initial resection, the patient stated that the persistent gross hematuria had returned. Cystoscopy showed a 3-cm tumor in the bladder dome. Biopsy demonstrated high-grade papillary UCC, this time with muscle invasion. The prior site of resection was negative for UCC (UTMC laboratories). Treatment options included RC, ileal conduit with transplant ureteral anastomosis with allograft pancreatectomy, and TMT. Immunotherapy with pembrolizumab was considered; however, the oncology team felt the risk of infection with conservative management was too high. The patient elected for TMT with complete TURBT followed by 6 rounds of chemoradiotherapy protocol (synchronous intensity-modulated radiation followed by mitomycin C and 5-fluorouracil). The patient completed the treatment course without associated adverse effects.

11 months post-initial resection, cystoscopy reveals no new mucosal change. 14 months post-initial resection, cystoscopy reveals no papillary lesions, and urine cytology showed rare atypical urothelial cells. At 18 months, cystoscopy noted grade 1 trabeculation and an erythematous patch on the posterior trigone near the prior resection site with a suspicious lesion. 2 deep biopsies at the suspicious erythematous site showed multiple fragments of mostly denuded rare, atypical urothelial cells with enlarged hyperchromatic nuclei in a background of inflammation and fibrosis. Biopsy from the resection scar was also negative for carcinoma recurrence.

At 22 months, cystoscopy with random site biopsy within an area of mucosal erythema showed focal carcinoma in situ without invasion. Immunohistochemistry analysis of the biopsy stained positive for cytokeratin 20 (CK 20). Biopsy of the prior resection site showed no evidence of dysplasia or cellular atypia (UTMC laboratories). At 31 months, cystoscopy identifies no new suspicious lesions to bladder mucosa. Patient defers biopsy. At 32 months, the patient presents to the ER in hypertensive crisis, discharged without medication changes. Etiology is not elucidated. At 41 months, cystoscopy showed grade 1 trabeculation of the bladder wall with patchy mucosal erythema. No bladder masses were grossly identified. Prior pathology results are discussed, but the patient defers repeat biopsy as he does not feel it is necessary.

46 months post-initial resection, the patient states that he had gross hematuria and UTI after last office cystoscopy, treated by PCP. Repeat cystoscopy shows no suspicious lesions and continued grade 1 trabeculation of the bladder wall with patchy mucosal erythema. 49 months post-resection, cystoscopy again showed no suspicious lesions, and 2 random biopsies with 1 deep biopsy showed no evidence of dysplasia or atypia (Mayo Clinic Laboratories and UTMC laboratories both report). Urine cytology is again indicative of high-grade urothelial carcinoma, with numerous tumor cells arranged as single cells in small groups with high nuclear to cytoplasmic ratio, irregular nuclear membranes, and hyperchromatic nuclei indicative.

At 55 months, a biopsy of a random site showed benign urothelial mucosa without evidence of carcinoma recurrence. Additional prostatic urethral biopsy showed benign urothelium and prostatic tissue, and CT scan of the abdomen and pelvis was done, none of which showed evidence of carcinoma recurrence. However, urine cytology still showed high-grade UCC. For the duration of the patient's cancer workup and treatment, renal and pancreatic function remained normal without evidence of rejection or dysfunction.

## 3. Discussion

It is well established that solid organ transplants increase the risk of future malignancy [7]. De novo malignancy in transplant patients has a worse prognosis compared to nontransplanted patients [8]. Kidney transplant is associated with increased risk for bladder cancer, presenting more aggressively and in advanced stages compared to nontransplanted patients [7, 9]. The most common presentation of bladder cancer post-transplantation is gross hematuria, warranting immediate workup, as in our patient [3].

Currently, no specific treatment guidelines exist for patients with pancreatic-kidney transplant patients that develop bladder cancer [1, 3]. The two most common treatment options for bladder cancer in the general population are RC or bladder TMT [10, 11]. For MIBC, the preferred treatment is RC with urinary diversion [11, 12]. For patients with MIBC that are medically unfit or unwilling to undergo aggressive treatment with RC, TMT is the most studied conservative alternative [2].

TMT consists of complete TURBT followed by chemotherapy and radiation [4, 10] which evaluated the outcomes and the overall survival rates in 160 patients with bladder cancer treated with either RC, TURBT, TMT, or palliative care with chemotherapy and radiation. The study found no difference in treatment outcomes for advanced UCC with RC and TURBT for patients over 76 years old; however, those under the age of 76 with treated with RC had increased disease-specific survival without recurrence [10]. Notably, none of these patients had history of SOT which may have altered treatment without chemoradiotherapy restrictions [10]. Another study on patients with MIBC found worse median overall survival with TMT compared to RC [13]. In our patient, it was decided not to perform RC to prevent compromise of the pancreatic transplant. However, there are potential risks to this conservative management of the pancreatic graft such as infection, radiation damage, cancer progression, or metastasis [14]. Additionally, immunotherapy with pembrolizumab was deemed to have too high a risk of infection by the oncology team and the patient refused BCG therapy.

To our knowledge, this is the first case of MIBC in a SPK transplant patient treated with TMT. Transplanted pancreatic exocrine secretion can be drained either enterically or through the bladder; however, bladder drainage is preferred to allow monitoring for pancreatic graft rejection through urine protein analysis [15]. Our patient with SPK transplant with pancreatic-bladder anastomosis developed MIBC. Treatment with TMT was preferred as RC risked compromising the pancreatic transplant. The patient had repeat negative cystoscopies after the tumor resections; thus, consolidative chemoradiotherapy with synchronized intensity-modulated radiation followed by mitomycin c and 5-FU was initiated. The chemotherapy regimen of 5-fluorouracil and mitomycin was chosen to avoid the risk of nephrotoxicity associated with standard cisplatin therapy [4].

## 4. Conclusion

Although SOT increases the risk of bladder cancer, it is still rare. Our patient is 55 months status post SOT without biopsy-proven recurrence. Due to the limited number of patients with history of pancreatic and kidney transplant who develop bladder cancer, there are no specific management guidelines for MIBC. This case demonstrates that trimodal therapy and bladder preservation in recipients of pancreas-kidney transplants may be safe and feasible with close follow, although further studies are needed.

## Data Availability

The data used to support the findings of this study are included within the article.

## Abbreviations

CT: Computed tomography
MIBC: Muscle-invasive bladder cancer
RC: Radical cystectomy
SPK: Simultaneous pancreas kidney
TURBT: Transurethral resection of bladder tumor
TMT: Trimodal therapy
UCC: Urothelial cell carcinoma.
